# Exercise as a Synchronizer: Effects on Circadian Re‐Entrainment of Core Body Temperature and Metabolism Following Light–Dark Cycle Inversion in Mice

**DOI:** 10.1111/jpi.70057

**Published:** 2025-05-13

**Authors:** Andrea Michele Ciorciari, Emanuel Irizarry, Angela Montaruli, Katja A. Lamia

**Affiliations:** ^1^ Department of Biomedical Sciences for Health University of Milan Milan Italy; ^2^ Department of Molecular and Cellular Biology Scripps Research La Jolla CA USA

**Keywords:** biological clock, body temperature, circadian rhythm, exercise, light, metabolism, mice

## Abstract

Core body temperature (CBT) is a crucial marker of circadian synchrony, reflecting behavioral, metabolic, and environmental adaptations. Disruptions to CBT rhythms, as seen in shift workers or jetlag, indicate desynchronization and can lead to significant health consequences. Exercise is a potent non‐photic zeitgeber that may help align circadian rhythms with external cues, but its role in re‐entrainment following abrupt phase shifts remains unclear. This study investigated whether voluntary exercise accelerates the re‐entrainment of CBT and metabolic rhythms in mice subjected to a 12‐h light‐dark cycle inversion (LDI). Fifteen C57BL/6 J mice underwent LDI and were divided into two groups. Mice in the control (CTRL) group remained sedentary throughout the experiment while mice in the other group were provided running wheels for 2 weeks after LDI. CBT was continuously monitored using implanted telemetric capsules and metabolic parameters were assessed before and 2 weeks after LDI. Mice that had access to running wheels (RW mice) initially displayed a greater disruption of CBT rhythmicity following LDI, suggesting unstructured physical activity may temporarily exacerbate misalignment, acting as a conflicting signal. Despite this, exercise accelerated recovery, as the phase of the CBT rhythm in RW mice re‐aligned to the new light‐dark cycle faster than that of the CTRL mice did. The phase of VO₂ rhythms in RW mice also showed trends toward faster realignment. These findings highlight the dual role of exercise as a zeitgeber, capable of both disrupting and accelerating circadian realignment depending on timing. Voluntary exercise may thus serve as an effective intervention to restore circadian synchrony and metabolic homeostasis in individuals experiencing circadian disruptions.

**Abbreviations**
AMP: Amplitude
ANOVA: Analysis of Variance
CBT: Core Body Temperature
CLAMS: Comprehensive Laboratory Animal Monitoring System
CTRL: Control
IQR: Interquartile Range
LDI: Light‐Dark Cycle Inversion
MESOR: Midline Estimating Statistic of Rhythm
PHI: Acrophase
PR: Percentage of Rhythm
PRC: Phase Response Curve
RER: Respiratory Exchange Ratio
RW: Running Wheel
SCN: Suprachiasmatic Nucleus
SPSS: Statistical Package for the Social Sciences
VO₂: Oxygen Consumption
VCO₂: Carbon Dioxide Production
ZT: Zeitgeber Time

## 1 Introduction

Several physiological processes are controlled by an endogenous biological clock, which is synchronized by external cues such as light and temperature [1] and regulates circadian rhythms. The suprachiasmatic nucleus (SCN) in the hypothalamus is the central pacemaker that orchestrates these daily oscillations. It receives light afferents from the retina and influences various bodily functions including metabolism [2], body temperature [3], and hormone secretion [4]. Physical activity is a potent modulator of circadian rhythms and has been shown to influence the synchronization, amplitude, and robustness of these cycles [5] by acting on many physiological factors, such as core body temperature (CBT) and metabolism. CBT is a reliable marker of circadian rhythms, reflecting the oscillatory nature of metabolic processes [3].

The relationship between exercise, CBT, circadian rhythms, and metabolism is complex and multifaceted, with each factor influencing and being influenced by the others. Among these, physical activity is a behavioral factor that is relatively easier to modulate, can positively impact metabolism [6], and facilitates the entrainment and synchronization of temperature and metabolic rhythms [7], with significant health implications. However, the effects of exercise on the re‐entrainment of circadian rhythms following disruptions, such as a light/dark (LD) inversion, remain less understood. This area of study is particularly relevant for understanding how organisms adapt to changes in their environment that affect *zeitgebers* (zeit = time, geber = giver, synchronizers). For example, shift workers and individuals with *social jetlag* [8] experience altered zeitgeber exposures and are at a higher risk for metabolic disorders and cancer [9, 10, 11]. Furthermore, insights into the readjustment of biological rhythms have implications for exercise physiology and performance, particularly for athletes who undergo transcontinental flights [12, 13]. Investigating the adaptations and re‐entrainment of organisms after significant disruptions can therefore provide valuable knowledge for improving health outcomes and performance.

In this study, we aim to determine whether physical activity alters the re‐entrainment of CBT in mice subjected to a light‐dark inversion (LDI, e.g. a 12‐h phase shift) compared to sedentary controls. Additionally, we evaluate metabolism through measurements of oxygen consumption and carbon dioxide production to provide a comprehensive overview of the physiological adaptations to LDI and physical activity.

## 2 Materials and Methods

All animal care, treatments, and experiments were overseen and approved by The Scripps Research Institute IACUC (*Institutional Animal Care and Use Committee*) under protocol #10‐0019.

### 2.1 Experimental Design

**Figure 1:**
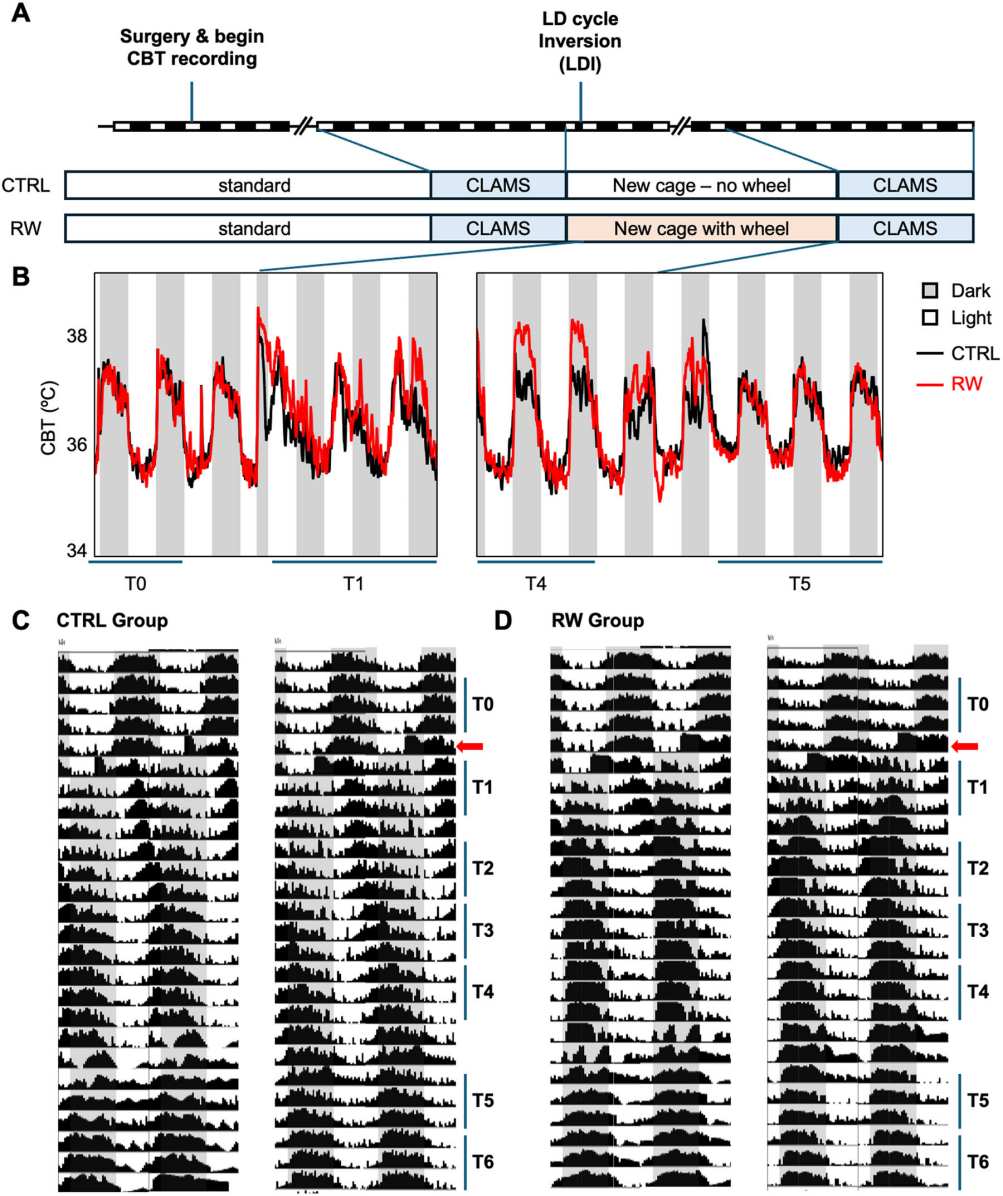
Core body temperature (CBT) rhythms before and after light‐dark inversion. (A) Graphical representation of the experimental timeline. A few weeks after surgical implantation of temperature‐monitoring capsules, mice were placed in CLAMS cages for 1 week to conduct baseline analysis. During this period, their baseline Core Body Temperature (CBT) rhythm (T0) was recorded. Following a light‐dark cycle inversion (LDI), half of the mice were transferred to running wheel (RW) cages. During this second week, which included T1 (the first 3 days after the inversion) and T2 (the fifth, sixth, and seventh days), the mice's readjustment in CBT was studied. Moreover, additional timepoints (T3, T4, T5 and T6) were evaluated to study the long‐term readjustment. Two weeks after the LD inversion and baseline CLAMS analysis, all mice were returned to metabolic cages for follow‐up CLAMS assessment. NOTE: The 2 days between T4 and T5 were excluded from the analysis because they corresponded to the period when the mice were transferred from their home cages to the metabolic cages, introducing a significant confounding factor. (B) CBT rhythms in CTRL (black) and RW (red) mice surrounding major transitions in the experimental design. Data represent the mean for 7 or 8 animals per group. Shaded rectangles represent the times when lights were on (white, light) or off (gray, dark) respectively. (C, D) Double‐plotted thermograms depicting CBT data in 5‐min bins, where the height of the black vertical lines corresponds to the body temperature. Each horizontal line represents two consecutive days with the second day repeated at the start of the line below. Representative thermograms are shown for two mice in the CTRL group (*C*) and two mice in the RW group (*D*).

A graphical representation of the experimental timeline is displayed in Figure 1A.

Fifteen male C57BL/6 J (weight = 26.67 ± 1.45 g) mice were bred at The Scripps Research Institute vivarium with ad libitum access to water and chow and regular LD cycle (lights on at 03:00 a.m., ZT0; lights off at 03:00 p.m., ZT12). Luminosity was measured with a Sekonic C‐7000 Spectrometer color meter. For collecting the spectral data, the room light was turned off and each controlled light enclosure was open one by one so that the only light available came from the lightbulb inside the boxes. The light intensity and color temperature measured 827–1240 lux and 3981–4067 K, respectively (Supporting Figure S1). All mice were then surgically implanted with intraperitoneal temperature‐recording devices, which recorded CBT for the whole duration of the study. One month after the surgery, the mice were 4 months old, and the experimental protocol began. The mice were weighed and transferred to metabolic cages designed to evaluate oxygen consumption and other metabolic parameters, which will be detailed further in this section. After 1 week of metabolic recording, the LD cycle was reversed (03:00 a.m. on – 03:00 p.m. off → 03:00 a.m. off – 03:00 p.m. on). Eight mice were transferred to cages equipped with a running wheel (RW group), while the remaining seven mice were housed in regular cages (CTRL group), so that the only difference between groups was the presence or absence of a running wheel. Two weeks later, all mice were returned to the metabolic cages for follow‐up analysis.

### 2.2 Temperature

CBT was recorded throughout the study using specialized capsules (Anipill®, BodyCap, Hérouville Saint‐Clair, France) that were implanted intraperitoneally in all mice. These devices were programmed to record temperature every 5 min. The recorded data were transmitted in real‐time to a monitor (Anilogger®, BodyCap, Hérouville Saint‐Clair, France), which was subsequently used to download the raw data for analysis.

### 2.3 Metabolism

The metabolism of the mice was evaluated by the *Comprehensive Laboratory Animal Monitoring System* (CLAMS, Columbus Instruments, Columbus, OH, USA), consisting of a system of metabolic cages able to measure simultaneously various metabolic parameters, such as oxygen consumption (*VO*_*2*_) and carbon dioxide production (*VCO*_*2*_). Food and water consumption, as well as locomotor activity levels using infrared lights, were recorded while the mice were in the metabolic cages.

### 2.4 Statistical Analysis

Measurements of CBT and of metabolic parameters in mice housed with access to running wheels (RW mice) were compared to those from mice housed without running wheels (control, CTRL mice). The normality of the distribution of the data was assessed by Shapiro‐Wilk Test. Levene's Test was used to evaluate the homogeneity of variance of the data. Repeated Measures ANOVA was used to compare the groups at three timepoints: *Time 0* (T0, which included either 3 or 7 days, according to the type of analysis, before LDI), *Time 1* (T1, the first 3 days after LDI), and *Time 2* (T2, the 5th, 6th, 7th days after LDI) in the temperature data, to compare the baseline (T0) with the most disrupted phase (T1) and the early readjustment phase (T2). Repeated Measures ANOVA was used also to compare RW and CTRL mice at baseline (before LDI) as well as across successive 3‐day intervals following Time 2 (T3 through T6, as shown in Figure 1C and 1D and Supporting Figure S3A) in the CBT data and after 2 weeks in the CLAMS data. If necessary, post‐hoc analyzes (Bonferroni adjusted) were performed. The two groups did not differ in terms of body weight (CTRL = 26.3 g ± 1.6 g; RW = 26.7 g ± 1.3 g; *p* = 0.35), therefore it was not included in the analysis as a covariate. The effect size was quantified according to Cohen [14]. The statistical analyzes were performed using SPSS Statistics version 28 (IBM SPSS Statistics for Windows, Armonk, NY, USA: IBM Corp), GraphPad Prism version 10.0.0 (GraphPad Software, Boston, USA), or in RStudio (Posit, Boston, USA; code available in GitHub repository KatjaLamia/Ciorciari_LDI), setting the statistical significance to α = 0.05.

### 2.5 Rhythm Analysis

The chronobiological analysis of the studied variables’ rhythms was performed using two methods. First, we used *Cosinor* analysis [15] to determine the *Percentage of Rhythm* (PR), the *Midline Estimating Statistic of Rhythm* (MESOR) and the *Amplitude* (AMP). PR represents the proportion of the total variance accounted for by the model fitted to the data. Essentially, it indicates how well the model fits the data. A higher PR suggests a tighter fit, indicating stronger rhythmicity. Conversely, a lower value implies the presence of noise and irregularities, indicating weaker rhythmicity. MESOR is the rhythm‐adjusted mean around which the rhythm oscillates over a specific period. AMP measures one half of the extent of variation in the rhythm. Therefore, it is the difference between the peak and the MESOR, and a high AMP indicates a strong and robust rhythm. The *acrophase* (PHI), which indicates the phase of the rhythm where the highest point occurs, was also investigated. The study of the rhythm was made with RStudio (Posit, Boston, USA), with the package for the *Cosinor* analysis *CatKIT* [16]. As a complement to the cosinor analysis, we estimated CBT and metabolic parameters rhythmicity without fitting to an idealized sinusoidal model. We used auto‐correlation to estimate *period*, which is the frequency with which the phenomenon occurs, cross‐correlation to determine the *phase angle*, which is the time difference between a rhythm and an external cue (relatively to the light cycle), and calculated averages for each parameter during the light and dark phases using standard procedures. Notably, acrophase estimations are based on a model assuming a fixed 24‐h period, whereas the phase angle is calculated from cross‐correlation analysis between the rhythmic variable of interest (CBT in this case) and the timing of light exposure (defined as a binary ON/OFF in this case). This method makes no assumptions about period length in the CBT data. We calculated phase shifts by subtracting either the acrophase (from Cosinor analysis) or the *phase angle* (from cross‐correlation analysis) of the baseline rhythm from that measured at multiple times after LDI. Additional detail is provided in Supporting Figure S2 and all code used for the analysis is available in GitHub repository KatjaLamia/Ciorciari_LDI.

## 3 Results

### 3.1 Core Body Temperature

At baseline (‘time 0’ or T0), when housed in standard lighting conditions (lights on at 03:00 a.m., ZT0; lights off at 03:00 p.m., ZT12), we measured the expected physiological circadian oscillation in CBT in c57BL6 male mice (Figure 1B‐D and Supporting Figure S3). Following LDI, the CBT rhythm was disrupted, as reflected by a decreased fit to an idealized sinusoidal rhythm (lower PR) and reduced amplitude in the days immediately following the shift (Table 1). These findings indicate that such a large phase shift in light exposure timing produced the expected disruption of circadian synchrony among physiological rhythms. Four days after the LDI (referred to as ‘time 2’ or T2), the CBT rhythmicity was restored. To determine whether physical activity alters the readjustment following a phase shift, we introduced running wheels to a separate group of mice enabling them to be physically active during 2 weeks of recovery from the light‐dark inversion. Hereafter, we refer to mice that were provided running wheels as the RW group and those that were housed in similar cages without running wheels as the control (CTRL) group.

**Table 1:** Core Body Temperature rhythm variables at T0, T1 and T2 from Cosinor analysis.

		CTRL		RW	
Timepoint	Variable	Mean	S.E.	Mean	S.E.
T0	CBT – PR (%)	50.41	4.16	47.65	3.36
	CBT – MESOR (°C)	36.38	0.05	36.35	0.04
	CBT – AMPLITUDE (°C)	0.79	0.04	0.79	0.07
	CBT – PHI (hh, mm)	15.86	0.26	16.86*	0.26
T1	CBT – PR (%)	28.40##	1.63	16.01* ^,^ ##	4.09
	CBT – MESOR (°C)	36.27	0.04	36.56	0.11
	CBT – AMPLITUDE (°C)	0.53#	0.03	0.47#	0.07
	CBT – PHI (hh, mm)	19.25###	0.54	21.82** ^,^ ###	0.54
T2	CBT – PR (%)	58.11++	5.28	65.55++ ^,^ ΨΨ	1.09
	CBT – MESOR (°C)	36.33	0.02	36.58	0.09
	CBT – AMPLITUDE (°C)	0.85++	0.08	1.23*** ^,^ +++ ^,^ ΨΨΨ	0.03
	CBT – PHI (hh, mm)	21.36++ ^,^ ΨΨΨ	0.41	23.25** ^,^ ΨΨΨ	0.41

* *p* < 0.05

** *p* < 0.01

*** *p* < 0.001 for RW versus CTRL.

^#^ *p* < 0.05,

^##^ *p* < 0.01,

^###^ *p* < 0.001 for T1 versus T0,

^++^ *p* < 0.01,

^+++^ *p* < 0.001 for T2 versus T1,

^ΨΨ^ *p* < 0.01,

^ΨΨΨ^ *p* < 0.001 for T2 versus T0.

PR: In the comparison between groups, CTRL mice showed a significantly higher *temperature PR* than RW mice at T1 (*p* = 0.01; F = 7.9; η^2^ = 0.44). In the comparison within groups, CTRL mice showed a significant decrease from T0 to T1 (*p* = 0.002; F = 29.7; η^2^ = 0.87) and an increase from T1 to T2 (*p* < 0.001; F = 29.7; η^2^ = 0.87). In contrast, RW mice showed a significant decrease from T0 to T1 (*p* < 0.001; F = 76.1; η^2^ = 0.94), an increase from T1 to T2 (*p* < 0.001; F = 76.1; η^2^ = 0.94) and an increase from T0 to T2 (*p* = 0.04; F = 76.1; η^2^ = 0.94).

MESOR: No statistically significant differences were observed either between or within groups.

AMP: In the comparison between groups, RW mice showed a significantly higher *temperature Amplitude* than CTRL mice at T2 (*p* < 0.001; F = 22.1; η^2^ = 0.67). In the comparison within groups, CTRL mice showed a significant decrease from T0 to T1 (*p* = 0.03; F = 10.2; η^2^ = 0.7) and an increase from T1 and T2 (*p* = 0.003; df = 2; F = 10.2; η^2^ = 0.7). In contrast, RW mice showed a significant decrease from T0 to T1 (*p* = 0.01; df = 2; F = 55.5; η^2^ = 0.93), an increase from T1 to T2 (*p* < 0.001; F = 55.5; η^2^ = 0.93) and an increase from T0 and T2 (*p* < 0.001; F = 55.5; η^2^ = 0.93).

PHI: In the comparison between groups, a statistically significant difference emerged at T0 (*p* = 0.02; F = 7.44; η^2^ = 0.43), at T1 (*p* = 0.007; F = 11.44; η^2^ = 0.53) and at T2 (*p* = 0.008; F = 0.52; η^2^ = 0.52). In the comparisons within groups, CTRL mice showed a significant difference in the *temperature Acrophase* from T0 to T1 (*p* < 0.001; F = 219.43; η^2^ = 0.98), from T1 to T2 (*p* = 0.007; F = 219.43;; η^2^ = 0.98) and from T0 to T2 (*p* < 0.001; F = 219.43; η^2^ = 0.98). In contrast, RW mice showed a significant difference from T0 to T1 (*p* < 0.001; F = 326.57; η^2^ = 0.99), from T0 to T2 (*p* < 0.001; F = 326.57; η^2^ = 0.99), but no significant differences from T1 to T2.

Mice in the RW group exhibited a similar response to LDI as the CTRL group did, with their CBT rhythmicity less robust at T1, and restored by T2 (Figure 1B‐D, Supporting Figure S3 and Tables 1 and 2). However, differences from the CTRL group were observed across the different phases of the experiment. Specifically, at T1, CBT for RW mice exhibited a significantly lower rhythmicity (PR) compared to CTRL mice, indicating a more pronounced disruption of the CBT rhythm (Table 1). Despite this greater disruption at T1, by T2, RW mice exhibited a higher PR and AMP than CTRL mice (Table 1).

**Table 2:** Core body temperature rhythm variables from autocorrelation and cross‐correlation.

		CTRL		RW	
Timepoint	Variable	Mean	s.e.	Mean	s.e.
T0	CBT – Period (hrs)	24.01	0.12	24.01	0.12
	CBT – Phase angle (hrs)	11.81	0.22	11.75	0.17
T1	CBT – Period (hrs)	24.97	0.19	24.67	0.20
	CBT – Phase angle (hrs)	4.08###	0.19	5.38* ^,^ ###	0.43
T2	CBT – Period (hrs)	25.04	0.22	24.38	0.28
	CBT – Phase angle (hrs)	7.86##	0.55	9.62#	0.59
T3	CBT – Period (hrs)	24.35	0.22	24.29	0.18
	CBT – Phase angle (hrs)	10.53##	0.45	11.38#	0.40
T4	CBT – Period (hrs)	24.14	0.09	23.93	0.17
	CBT – Phase angle (hrs)	11.90#	0.27	12.33	0.26
T5	CBT – Period (hrs)	24.18	0.13	23.68	0.31
	CBT – Phase angle (hrs)	12.57	0.15	12.38	0.14
T6	CBT – Period (hrs)	23.64	0.12	23.48	0.24
	CBT – Phase angle (hrs)	12.35	0.22	12.46	0.08

* *p* < 0.05 for RW versus CTRL.

^#^ *p* < 0.05,

^##^ *p* < 0.01,

^###^ *p* < 0.001 in comparison to the previous Timepoint (e.g., T1 vs. T0, T4 vs. T3).

Period: In the comparison between groups, there were no statistically significant differences. In the comparison within groups, RW mice showed a significant increase from T0 to T1 (*p*adj = 0.045). There is a clear trend in the CTRL group, and although the apparent effect size is larger, it is not statistically significant (*p* = 0.15) because the change in period for individual mice is less consistent (Supporting Figure S4). All *p* values included here are adjusted for multiple comparisons. For simplicity, we will refer to adjusted *p* values hereafter as “*p*”.

Phase: In the comparison between groups, a statistically significant difference emerged at T1 (*p* = 0.03). In the comparisons within groups, CTRL mice showed a significant difference in the *phase angle* from T0 to T1 (*p* < 0.0001) and T2 (*p* = 0.006), and from T1 to T2 (*p* = 0.007), T3 (*p* = 0.0004), T4 (*p* < 0.0001), T5 (*p* < 0.0001), and T6 (*p* < 0.0001), and from T2 to T3 (*p* = 0.007), T4 (*p* = 0.006), T5 (*p* = 0.003), and T6 (*p* = 0.004), and from T3 to T4 (*p* = 0.04). RW mice showed a significant difference in the *phase angle* from T0 to T1 (*p* < 0.0001), and from T1 to T2 (*p* = 0.01), T3 (*p* = 0.0007), T4 (*p* = 0.0001), T5 (*p* = 0.0005), and T6 (*p* = 0.0001), and from T2 to T3 (*p* = 0.05).

**Figure 2:**
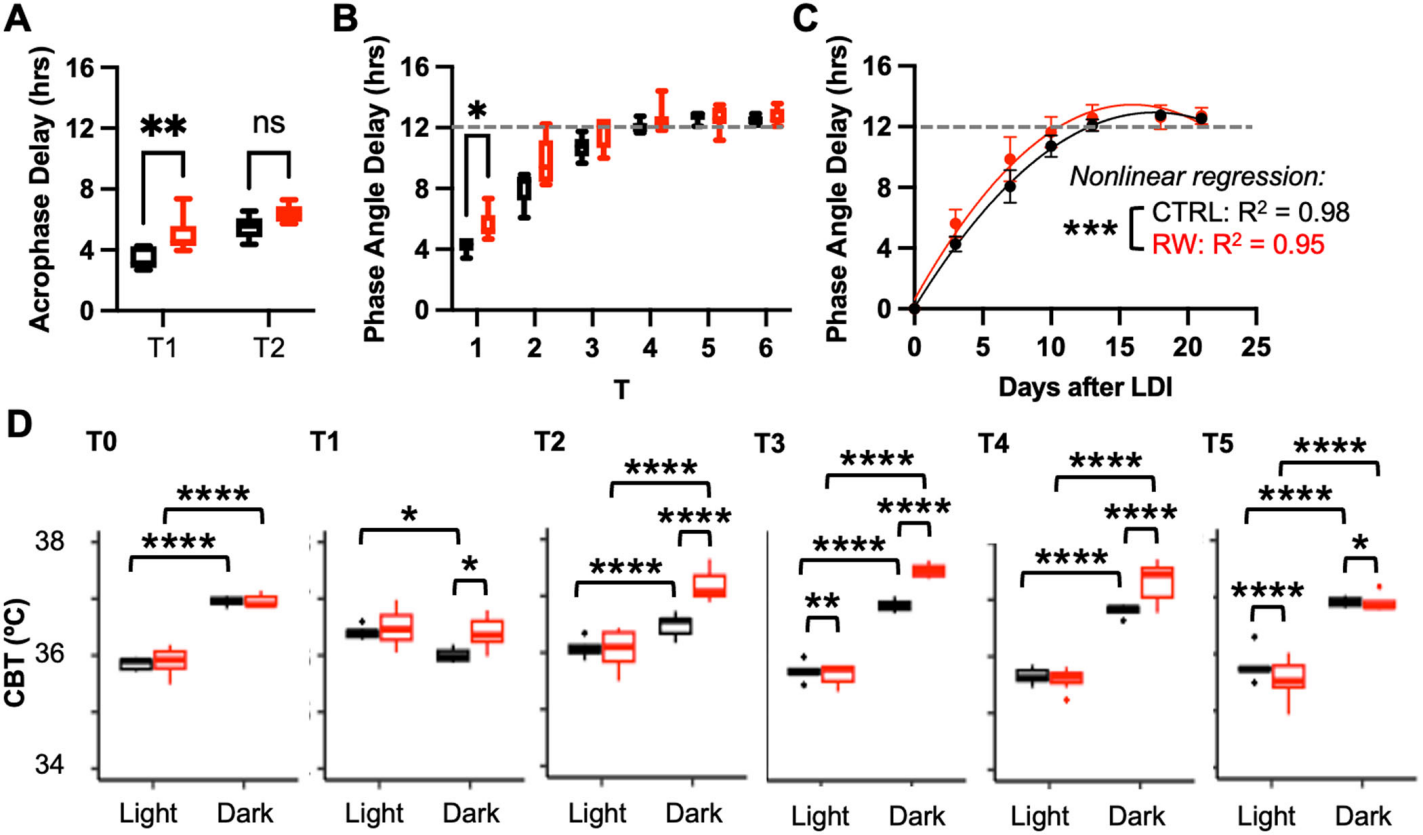
Physical activity accelerates re‐entrainment of CBT after light‐dark inversion. (A–C) Phase shifts calculated from CBT data for CTRL (black) and RW (red) mice by Cosinor analysis (A) or the cross‐correlation method (B, C). (D) CBT during light and dark phases in CTRL (black) and RW (red) mice at the indicated time periods. In (A, B, D) boxplots depict the median and interquartile range (IQR); whiskers extend either to the minimum or maximum data point or 1.5*IQR beyond the box, whichever is shorter. *p < 0.05, **p < 0.01, ***p < 0.001, ****p < 0.0001 by two‐way ANOVA.

While mice in both groups fully recovered rhythmicity by T2, the phase of the rhythm had not fully realigned. Visual inspection of the CBT data represented as double‐plotted ‘thermograms’ suggests that realignment is accelerated in the RW group (Figure 1C–D). To achieve full readjustment following LDI, the phase of the CBT rhythm (either the *acrophase* or the *phase angle*) would need to shift by 12 h. In CTRL mice, the acrophase measured using Cosinor analysis had delayed by 3.4 h at T1 and 5 h by T2. In contrast, RW mice showed a significantly greater phase delay of 5.5 h at T1 and 6.4 h by T2, bringing them closer to alignment with the new LD cycle, likely due to the synchronizing effect of exercise (Figure 2A and Table 1). Evaluation of the phase shift by the cross‐correlation method also shows a faster readjustment to the new phase after LDI in RW mice (Figure 2B and C and Table 2). The observed faster adjustment of the CBT rhythm in the RW group is consistent with the well‐known synchronizing effect of exercise on circadian rhythms.

Estimating the period of body temperature rhythms using autocorrelation revealed dynamic period length adjustments from T1 to T5 as the animals adjusted to the new phase. Although the changes between individual phases (T1 through T5) did not reach statistical significance, the main effect of experimental phase was highly significant (*p* < 0.0001). Periods that temporarily exceeded 24 h likely reflect times at which phase is being delayed and those less than 24 h may be caused by overshooting the phase and responding to entraining signals to readjust. Greater variability in the period of the RW group in the later phases may reflect conflicting re‐entrainment signals emanating from the light and exercise phase response curves (Table 2 and Supporting Figure S4).

By measuring the average CBT during the light and dark phases, we found that CBT was significantly higher during the dark phase in all mice before the LDI and was not different between the CTRL and RW groups, as expected. Immediately after LDI (during T1), this pattern was disrupted. One week after LDI, at the end of T2, the significantly higher CBT during the dark phase was reestablished and the average CBT during the dark phase was significantly higher in the RW group (Figure 2D, T2 through T4). When running wheels were removed to transfer the mice to metabolic cages (Figure 2D, T5), the average CBT in the RW group was reduced, suggesting that the higher nighttime CBT in RW mice at T2 through T4 can be attributed to their higher physical activity.

### 3.2 CLAMS

**Figure 3:**
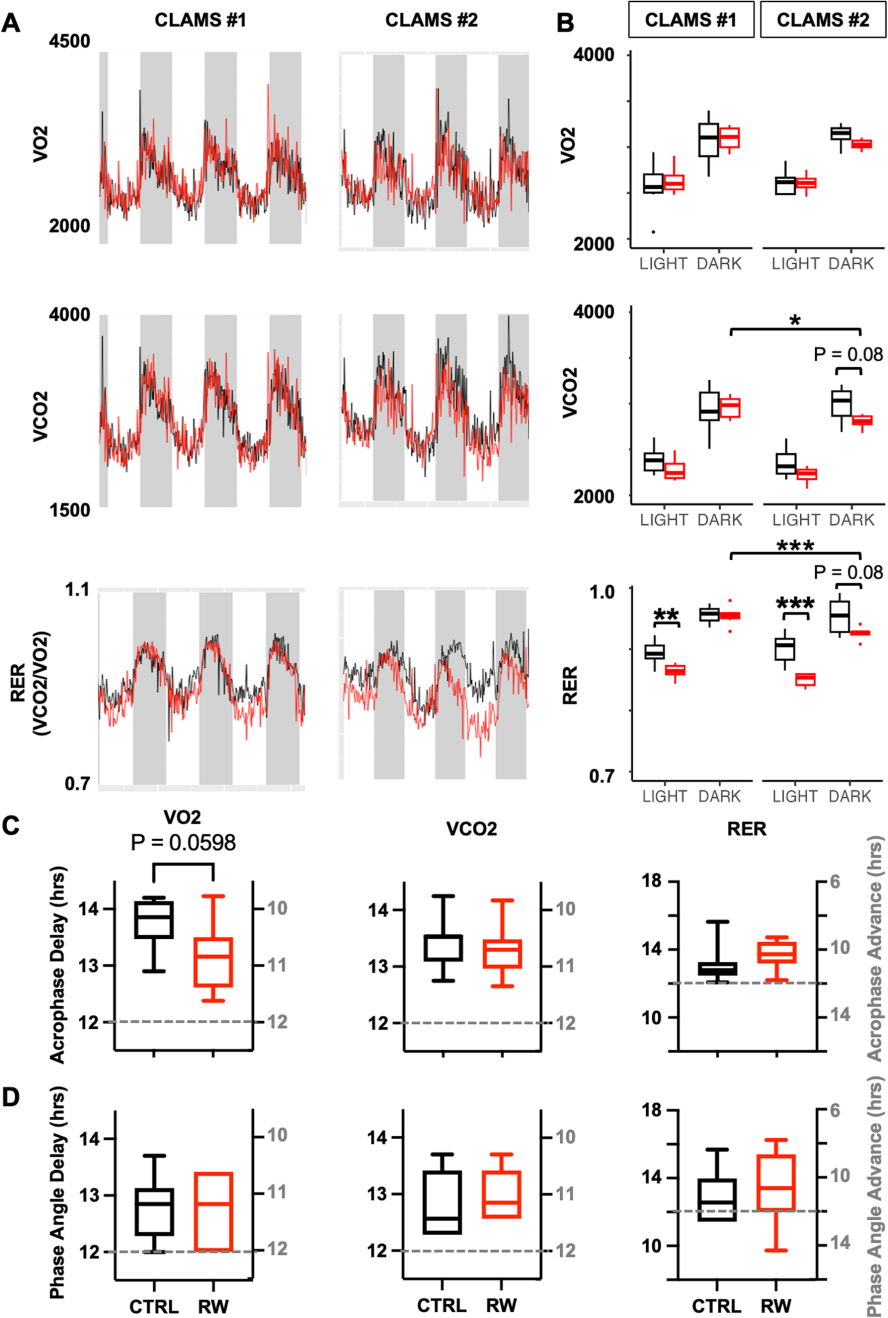
Physical activity alters metabolism during readjustment following light‐dark inversion. (A) VO_2_, VCO_2_ and RER rhythms of CTRL (black) and RW (red) mice before (CLAMS #1) and two weeks after (CLAMS #2) light‐dark inversion. Data represent the mean of 7 or 8 animals per group. (B) Mean VO2, VCO2, and RER during light (L) or dark (D) hours before (CLAMS #1) or two weeks after (CLAMS #2) light‐dark inversion. (C, D) Phase shifts calculated from CLAMS data for CTRL (black) and RW (red) mice using Cosinor (C) or cross‐correlation (D) analysis methods. In (B–D) boxplots depict the median and interquartile range (IQR); whiskers extend either to the minimum or maximum data point or 1.5*IQR beyond the box, whichever is shorter. *p < 0.05, **p < 0.01, ***p < 0.001 by two‐way ANOVA with Tukey's correction for multiple hypothesis testing.

No significant differences between RW mice and CTRL mice were observed from analysis of rhythmic parameters in CLAMS data measured before the experimental protocol began and 2 weeks after the LDI (Tables 3, 4). Within‐group analysis revealed a slight increase in rhythmicity for both oxygen consumption (VO_2_) and activity in both groups 2 weeks after LDI. RW mice also showed a higher amplitude of rhythmicity for both VO_2_ and activity during the second CLAMS test than in the test performed before LDI, likely due to their increased activity levels in the weeks leading up to the CLAMS analysis. Consistent with the expected impact of training, carbon dioxide production (VCO_2_) and respiratory exchange ratio (RER = VCO_2_/VO_2_) were lower in the dark phase in the RW group during the follow‐up period, indicating that they maintain improved fitness for at least 5 days without continuous access to running wheels (Figure 3A and B).

**Table 3:** Descriptive table of the metabolic values obtained from the CLAMS analysis at baseline and follow‐up in CTRL and RW mice.

		CTRL		RW	
Timepoint	Variable	Mean	S.E.	Mean	S.E.
BASELINE	FOOD INTAKE (g/day)	3.00	0.28	3.00	0.1
	VO_2_ – PR (%)	19.57	2.69	19.01	2.73
	VO_2_ – MESOR (mg/kg/hr)	2884.09	49.59	2687.33	178.04
	VO_2_ – AMPLITUDE (mg/kg/hr)	292.06	28.33	299.65	27.44
	VO_2_ – ACROPHASE (hh, mm)	19. 69	0.21	19.73	0.19
	VCO_2_ – PR (%)	39.31	2.02	31.76	3.71
	VCO_2_ – MESOR (mg/kg/hr)	2637.40	42.31	2467.62	194.76
	VCO_2_ – AMPLITUDE (mg/kg/hr)	474.81	23.45	408.79	32.24
	VCO_2_ – ACROPHASE (hh, mm)	19.93	0.2	19.88	0.18
	ACTIVITY – PR (%)	7.89	1.09	6.26	0.97
	ACTIVITY – MESOR	761.12	171.68	833.75	80.23
	ACTIVITY – AMPLITUDE	204.58	26.64	180.49	21.61
	ACTIVITY – ACROPHASE (hh, mm)	19.25	0.62	17.97	0.58
FOLLOW‐UP	FOOD INTAKE (g/day)	2.97	0.14	3.06	0.25
	VO_2_ ‐PR (%)	23.65#	2.00	27.60###	3.24
	VO_2_ – MESOR (mg/kg/hr)	2828.22	26.29	2691.13	190.44
	VO_2_ – AMPLITUDE (mg/kg/hr)	305.57	17.66	367.61	29.87
	VO_2_ – ACROPHASE (hh, mm)	9.42	0.16	8.89 *	0.15
	VCO_2_ – PR (%)	39.24	1.20	36.79	3.32
	VCO_2_ – MESOR (mg/kg/hr)	2519.64	28.78	2460.25	223.01
	VCO_2_ – AMPLITUDE (mg/kg/hr)	437.89	11.78	456.26	32.98
	VCO_2_ – ACROPHASE (hh, mm)	9.37	0.17	9.19	0.16
	ACTIVITY – PR (%)	12.85#	1.46	12.96##	2.69
	ACTIVITY – MESOR	725.27	135.73	807.34	88.36
	ACTIVITY – AMPLITUDE	225.51	23.16	235.22#	41.57
	ACTIVITY – ACROPHASE (hh, mm)	9.81	0.46	8.49	0.43

* *p* < 0.05; **p < 0.01; ***p < 0.001 for RW versus CTRL.

^#^ *p* < 0.05,

^##^ *p* < 0.01,

^###^ *p* < 0.001 for baseline (CLAMS Test #1) versus follow‐up (CLAMS Test #2).

VO_2_: In the comparison between groups, a statistically significant difference (*p* = 0.03; F = 6.1; η^2^ = 0.32) emerged at follow‐up in the *Acrophase*. In the comparison within groups, both CTRL (*p* = 0.04; F = 4.9; η^2^ = 0.28) and RW mice (*p* < 0.001; F = 25.1; η^2^ = 0.67) showed an increase in their *VO*_*2*_ *PR* between baseline and follow‐up. Moreover, CTRL mice showed no difference between baseline and follow‐up, while RW mice showed an increase in their *VO*_*2*_ *Amplitude* (*p* = 0.004; F = 12.2; η^2^ = 0.49).

VCO_2_: No significant differences were observed either between or within groups in *VCO*_*2*_.

Activity: In the comparison between groups, no significant differences emerged. In the comparison within groups, both CTRL (*p* = 0.03; F = 5.9; η^2^ = 0.31) and RW mice (*p* = 0.004; F = 12.3; η^2^ = 0.48) showed an increase in their *Activity PR* between baseline and follow‐up. Moreover, CTRL mice showed no difference between baseline and follow‐up, while RW mice showed an increase in their *Activity Amplitude* (*p* = 0.04; F = 4.7; η^2^ = 0.27).

**Table 4:** Metabolic rhythm variables from autocorrelation and cross‐correlation.

		CTRL		RW	
Timepoint	Variable	Mean	s.e.	Mean	s.e.
Baseline	VO_2_ – Period (hrs)	23.96	0.20	23.64	0.08
	VO_2_ – Phase angle (hrs)	11.54	0.18	11.78	0.11
	VCO_2_ – Period (hrs)	23.72	0.12	23.96	0.14
	VCO_2_ – Phase angle (hrs)	11.58	0.19	11.82	0.14
	Activity – Period (hrs)	23.68	0.22	23.96	0.71
	Activity – Phase angle (hrs)	11.54	0.14	10.89	1.25
Followup	VO_2_ – Period (hrs)	24.29	0.30	24.45	0.16
	VO_2_ – Phase angle (hrs)	12.30	0.16	12.55	0.13
	VCO_2_ – Period (hrs)	24.04	0.23	24.29	0.19
	VCO_2_ – Phase angle (hrs)	12.39	0.17	12.79	0.07
	Activity – Period (hrs)	25.05	0.43	24.77	0.87
	Activity – Phase angle (hrs)	12.55	0.20	10.56	0.98

Period: No statistically significant differences were observed either between or within groups.

Phase angle: No statistically significant differences were observed either between or within groups.

Because we did not monitor metabolic parameters for the first 2 weeks after the LD inversion, we can't determine whether circadian rhythms in metabolic parameters were progressively delayed like the CBT rhythms were or if they adjusted via progressive phase advances or some other pattern. Like we did for CBT, we used two methods to estimate phase shifts in metabolic parameters by comparing either the acrophase or the phase angle between baseline and follow‐up. While phase estimates were variable, neither CTRL nor RW mice appear to fully realign their metabolic rhythms to the new light‐dark cycle within 2 weeks (Figure 3C and D and Tables 3 and 4). Mice that were more physically active (RW mice) exhibited a significantly better realignment of the acrophase of their oxygen consumption (VO_2_) rhythm than the sedentary (CTRL) group (Figure 3C and Table 3). *Phase angle* estimation using the cross‐correlation method also indicated that neither the CTRL nor RW group had fully re‐aligned to the new LD cycle within 2 weeks, but there were no significant differences between CTRL and RW groups using this method.

## 4 Discussion

Core Body Temperature is a crucial marker for assessing how well an organism is synchronized to various zeitgebers, as it reflects behavioral, environmental, metabolic, and endocrinological factors [17, 18, 19], all of which are shaped by and contribute to circadian rhythms [20]. A disrupted circadian rhythm of body temperature is a symptom of desynchronization [3], a condition often observed in shift workers [21], which can lead to numerous medical issues [9]. Exercise plays a significant role in aligning circadian rhythms with zeitgebers, though the mechanisms through which it operates remain somewhat unclear. The aim of this study was to investigate whether voluntary exercise could accelerate the re‐entrainment of CBT and metabolism in mice after a LDI.

Our findings demonstrated that voluntary exercise, as expected, is associated with stronger CBT rhythms. RW mice exhibited higher PR and AMP at T2 compared to CTRL mice, and their PR and AMP were also elevated relative to their baseline (T0). Moreover, running wheel activity appears to accelerate the readjustment, as by T2 the acrophase in RW mice was closer to the expected target. This trend was observed not only in CBT, but in VO_2_ consumption as well. Interestingly, the RW group initially showed a more pronounced disruption (lower PR at T1) than the CTRL group, suggesting that physical activity may initially exacerbate circadian misalignment following a sudden environmental change. This observation highlights a critical consideration: while exercise is a potent zeitgeber, unstructured physical activity, like in our study where mice had continuous access to running wheels, can act as a conflicting signal, similar to providing constant light or food, thereby weakening its entraining effect.

Previous studies support this finding. Castillo et al. [22] reported that exercise at the “wrong” time (during the light phase) following LD inversion, with running wheels locked during the new dark period and available during the new light period, slowed the re‐entrainment process. Even after 2 weeks, many of the wheel‐restricted mice were still active predominantly during the light period. Similarly, Dallmann and Mrosovsky [23] found that nocturnal hamsters could be induced to run primarily during the light phase after a LDI, significantly slowing their re‐entrainment.

Although it is challenging to determine the exact role of exercise in facilitating re‐entrainment after a light shift, it is likely that structured exercise timing could aid the process. However, it is well established—and our study confirms—that physical activity can strengthen an organism's circadian rhythm under a normal LD cycle [24, 25, 26], and possibly accelerate its re‐entrainment. Additionally, Dallmann et al. [27] demonstrated that the absence of exercise, due to restricted wheel access, prevented mice from achieving light‐driven re‐entrainment even after 21 days of exposure to an inverted LD cycle.

Although exercise is a strong synchronizer, it is weaker than the photic zeitgeber [28, 29]. For instance, Sato and Yamanaka reported that under constant darkness, non‐photic entrainment of circadian rhythms through scheduled access to a running wheel took 49 days, underscoring the necessity of light for effective re‐entrainment.

A key challenge in interpreting our results is distinguishing true circadian re‐entrainment from potential masking effects due to exercise. To address this, we assessed both core body temperature and metabolism, as they provide complementary insights into circadian adaptation. While both are influenced by exercise, mice did not have access to the running wheel during measurements of metabolic parameters, reducing the direct impact of voluntary activity on all outcomes at that phase of the study. This setup helped differentiate longer‐term circadian adaptations from immediate exercise‐induced effects.

Strategically timing exercise to align with an individual's circadian rhythms may maximize its health benefits [30]. Exercise timed correctly could accelerate the re‐entrainment process and could be particularly beneficial in populations affected by circadian disruption, such as shift workers, who are prone to metabolic diseases due to insulin resistance [9]. Appropriately timed exercise might improve metabolic health markers in these populations. Gabriel & Zierath (2019) also suggest that increasing the amplitude of circadian rhythms could enhance tolerance to shift work.

Another group that may benefit from the synchronizing effects of exercise includes individuals experiencing *social jetlag* [8]. Studies have shown that those experiencing greater social jetlag tend to engage in less physical activity and perform worse in physical tasks [10, 31]. Conversely, in laboratory conditions, exercise has been shown to aid re‐entrainment in desynchronized mice [32], with clock gene expression in mice with ad libitum access to a running wheel suggesting a higher adaptive capacity to a new LD cycle. Moreover, wheel‐running seems to aid the disordered body clock in a mouse model that mimics social jet lag [33]. Oneda et al. showed that mice with access to running wheels exhibited faster synchronization of activity onset on weekdays, which had been delayed by social jet lag.

In terms of metabolism, our study did not find significant differences between the RW and CTRL groups in VO_2_, VCO_2_, activity, feeding, or drinking behaviors at baseline. Within‐group analyzes showed a slight increase in rhythmicity (PR) for both VO_2_ and activity in both groups at follow‐up, indicating stronger circadian rhythms in oxygen consumption and rest‐activity patterns. RW mice also showed higher VO_2_ AMP and activity AMP at follow‐up, likely due to their enhanced fitness levels during the study. Moreover, a significant difference in VO2 was observed at follow‐up between CTRL and RW mice, with the RW group showing an acrophase closer to the target, possibly indicating a faster re‐entrainment of metabolic processes as well. VO₂ and RER rhythms are typically in phase, but their re‐entrainment dynamics may be regulated differently. VO₂ is largely driven by activity levels and metabolic demand, while RER reflects substrate utilization, influenced by factors like energy balance, feeding behavior, and hormones. The faster VO₂ re‐entrainment in RW mice likely stems from increased activity accelerating metabolic adjustments. In contrast, the unchanged RER rhythm suggests that substrate utilization adapts more slowly. Additionally, since RER is the ratio of VCO₂ to VO₂, shifts in one variable (e.g., VCO₂) may not always result in significant RER changes, as the ratio can mask individual effects. Nevertheless, our study had limitations, particularly in terms of metabolic analysis. It would have been informative to assess metabolic rhythms immediately following the LD inversion, but the experimental setup did not allow continuous recording of metabolic data during wheel‐running.

In addition to its health implications, studying the effects of exercise on circadian re‐entrainment holds value for athletic performance. Jetlag and circadian misalignment can impair performance, and understanding how organisms readjust after major shifts in the light/dark cycle may help manage performance degradation following transmeridian flights [12].

A recent study [34] demonstrated that exercise performed before 4 p.m. induces phase‐peak advances, while exercise after 10 p.m. leads to phase‐peak delays. However, future studies should further investigate the optimal timing of exercise for circadian re‐entrainment, focusing on structured interventions and refining the phase‐response curve to exercise. Research should also address the metabolic responses immediately after LD inversions to gain a more comprehensive understanding of how physical activity influences both circadian rhythms and metabolism. Also, introducing novel environmental stimuli such as new objects, either alongside or in place of running wheels, could impact CBT and metabolic regulation similarly. Additionally, investigations into the molecular pathways involved in exercise‐induced re‐entrainment could provide deeper insights into the interaction between clock genes and physical activity. Finally, given that corticosterone (the analog of cortisol in humans) plays a crucial role in circadian regulation, metabolism, and the stress response, it would be important to measure its secretion rhythm in study designs similar to this one. While C57BL/6 J mice do not produce melatonin [35], its role in circadian entrainment is well established in diurnal species, where it is regulated by the pineal gland and SCN. Given the reciprocal relationship between corticosterone and melatonin in humans, future studies should investigate how their rhythms interact during re‐entrainment, particularly in relation to rest‐activity cycles and sleep. Such studies could also focus on populations at risk for circadian misalignment, such as shift workers, people with social jetlag, and athletes, to determine the most effective exercise protocols for mitigating the adverse effects of circadian disruption.

## Author Contributions

Conceptualization: Andrea Michele Ciorciari, Katja A. Lamia. Methodology: Andrea Michele Ciorciari, Katja A. Lamia. Formal analysis: Andrea Michele Ciorciari, Katja A. Lamia. Investigation: Andrea Michele Ciorciari, Emanuel Irizarry. Resources: Katja A. Lamia. Data curation: Andrea Michele Ciorciari, Katja A. Lamia. Writing – original draft: Andrea Michele Ciorciari. Writing – review and editing: Katja A. Lamia, Angela Montaruli, Emanuel Irizarry. Supervision: Katja A. Lamia, Angela Montaruli. Project administration: Katja A. Lamia. Funding acquisition: Katja A. Lamia.

## Conflicts of Interest

Authors declare no conflicts of interest.

## Supporting information


**Supporting Figure S1. Related to Figure 1.** (A) Placement of the Spectrometer for data acquisition on each shelf of the compartments used in this study. (B) Detailed wavelength distribution and relative intensity of light measured on one of the four shelves as a representative example. (C) Maximum luminosity, correlated color temperature (CCT), and ultraviolet radiation measured on each shelf.

**Supporting Figure S2. Related to Figure 1.** (A) Graphical representation of the variables describing a rhythm. MESOR is the rhythm‐adjusted mean around which the rhythm oscillates over a specific period. Amplitude measures one half of the extent of variation in the rhythm. Therefore, it is the difference between the peak and the MESOR, and a high Amplitude indicates a strong and robust rhythm. Acrophase indicates the phase of the rhythm where the highest point occurs. The Period is the frequency with which the phenomenon occurs. Phase angle is the time difference between a rhythm and an external cue (relatively to the light cycle). (B) Phase shifts were calculated using either the difference between acrophases or the difference between phase angles in this study.

**Supporting Figure S3. Related to Figure 1.** (A) CBT data for representative individual mice. (B) CBT data (black trace) and sinusoidal fit curve (blue trace) for representative individual mice in the RW and CTRL groups. A close‐up on T1 and T2 (early readjustment) is shown. Days 1‐7 represent the period before the LD inversion (T0), where all the mice were housed in regular cages. P1, P2, and P3 (post 1, post 2, and post 3) correspond to T1, the first three days after the inversion. P5, P6, and P7 indicate T2, the fifth, sixth, and seventh days after the inversion.

**Supporting Figure S4. Related to Table 2.** (A,B) Period values determined by autocorrelation for individual mice throughout the experiment (*A*) or in experimental phases T0 and T1 showing the change for each animal (*B*). Black symbols and lines depict mice maintained in cages without running wheels throughout the experiment (CTRL); red symbols and lines depict mice that were provided running wheels after the light‐dark inversion (RW).

## Data Availability

All data are available in GitHub repository KatjaLamia/Ciorciari_LDI.
