# Supplementary figures and images for: Exercise as a Synchronizer: Effects on Circadian Re‐Entrainment of Core Body Temperature and Metabolism Following Light–Dark Cycle Inversion in Mice
Source: J Pineal Res. 2025 May 13;77(3):e70057. doi: 10.1111/jpi.70057 (PMC12070452; doi:10.1111/jpi.70057)

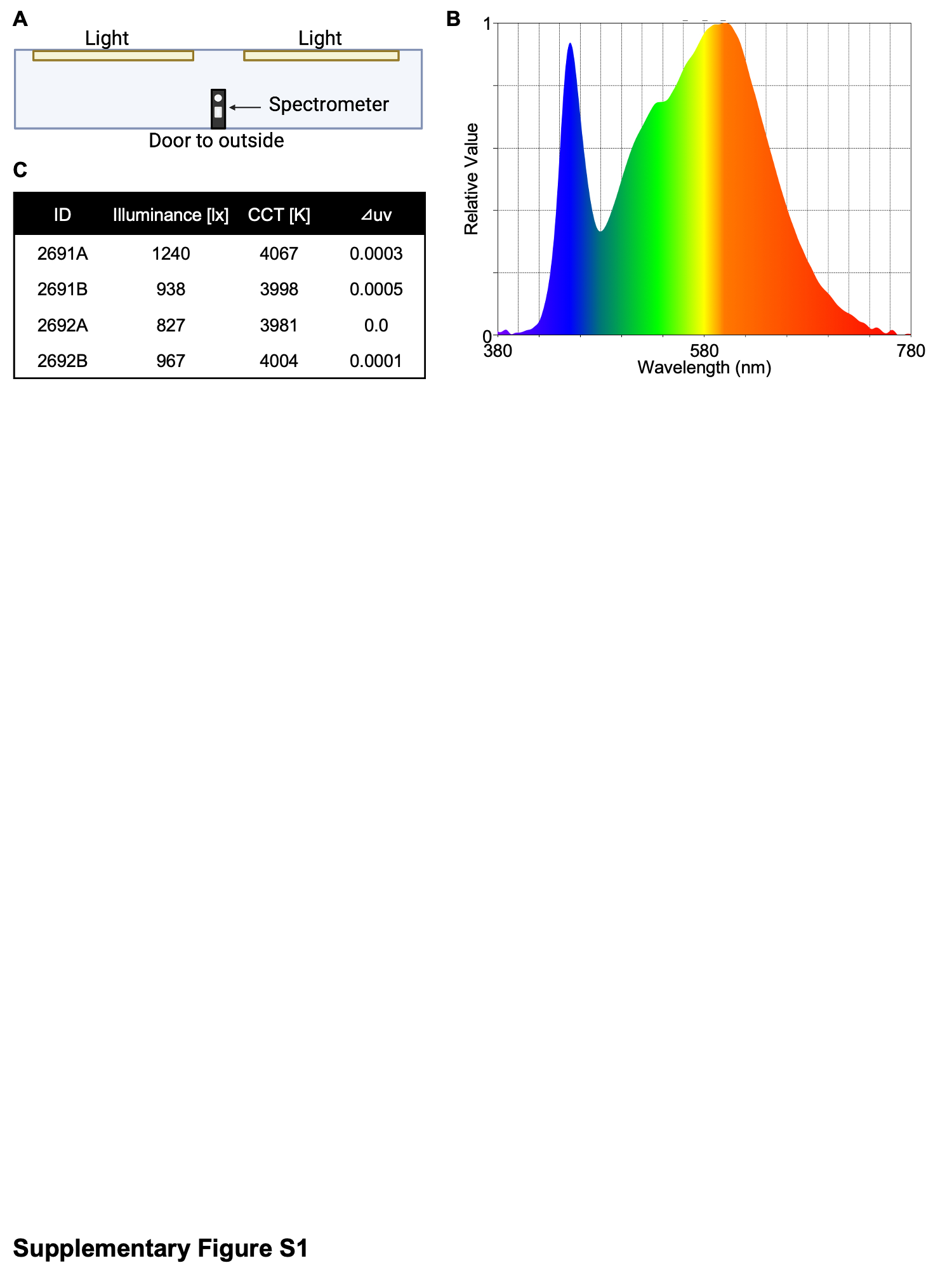

Supplement: Supplementary file 1 — Supporting Figure S1. Related to Figure 1. (A) Placement of the Spectrometer for data acquisition on each shelf of the compartments used in this study. (B) Detailed wavelength distribution and relative intensity of light measured on one of the four shelves as a representative example. (C) Maximum luminosity, correlated color temperature (CCT), and ultraviolet radiation measured on each shelf. [file JPI-77-e70057-s001.tiff]

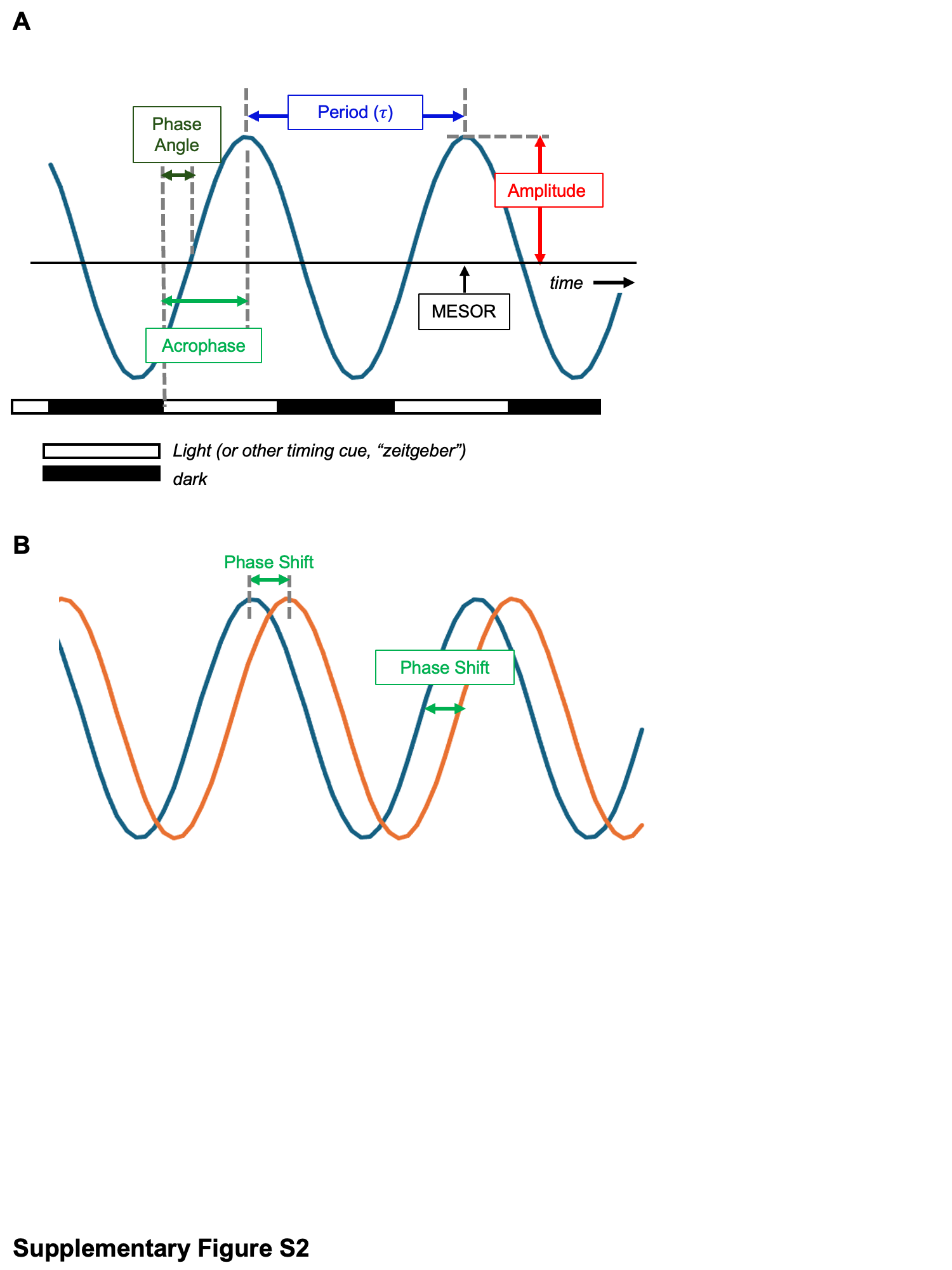

Supplement: Supplementary file 2 — Supporting Figure S2. Related to Figure 1. (A) Graphical representation of the variables describing a rhythm. MESOR is the rhythm‐adjusted mean around which the rhythm oscillates over a specific period. Amplitude measures one half of the extent of variation in the rhythm. Therefore, it is the difference between the peak and the MESOR, and a high Amplitude indicates a strong and robust rhythm. Acrophase indicates the phase of the rhythm where the highest point occurs. The Period is the frequency with which the phenomenon occurs. Phase angle is the time difference between a rhythm and an external cue (relatively to the light cycle). (B) Phase shifts were calculated using either the difference between acrophases or the difference between phase angles in this study. [file JPI-77-e70057-s003.tiff]

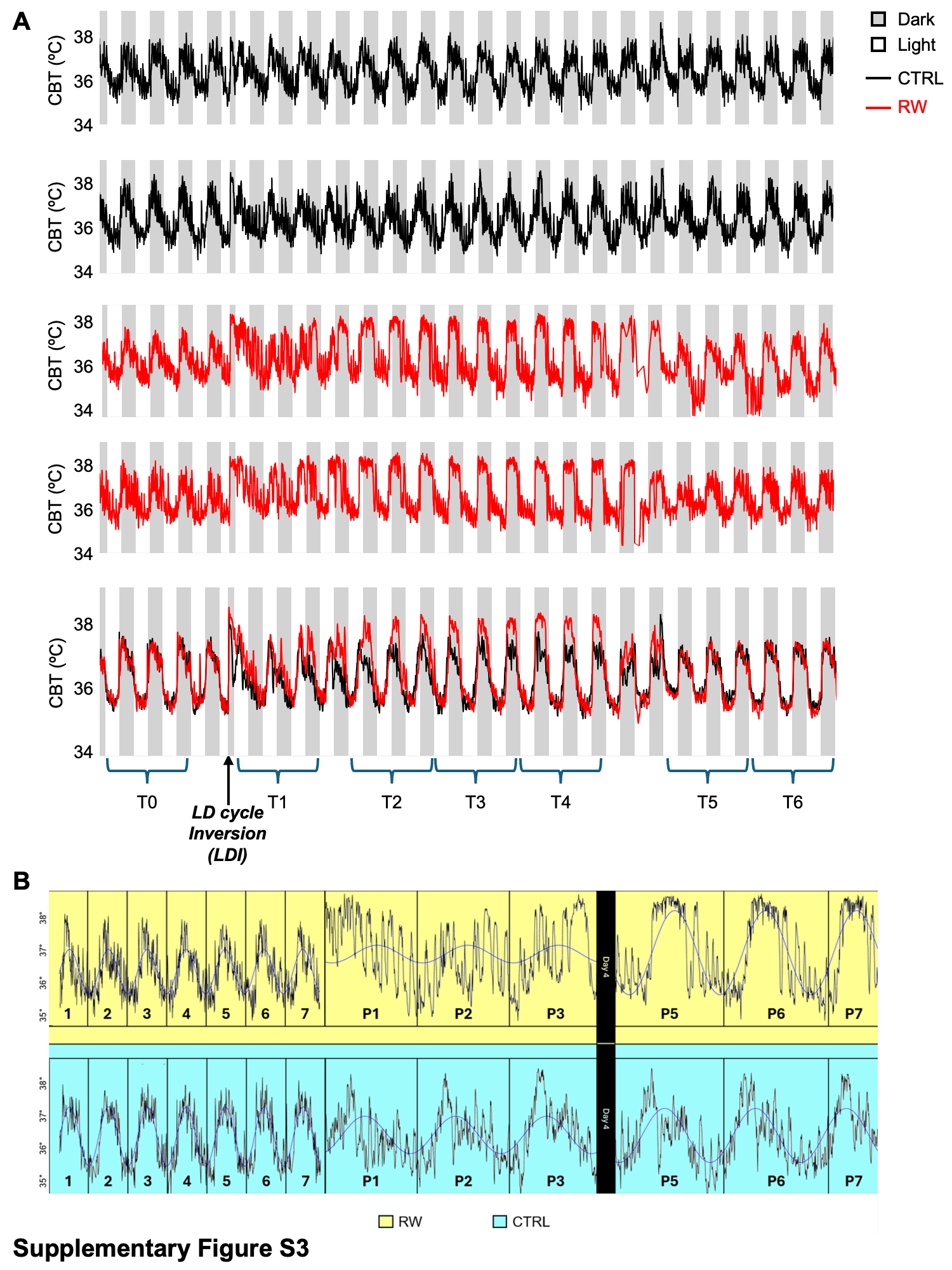

Supplement: Supplementary file 3 — Supporting Figure S3. Related to Figure 1. (A) CBT data for representative individual mice. (B) CBT data (black trace) and sinusoidal fit curve (blue trace) for representative individual mice in the RW and CTRL groups. A close‐up on T1 and T2 (early readjustment) is shown. Days 1‐7 represent the period before the LD inversion (T0), where all the mice were housed in regular cages. P1, P2, and P3 (post 1, post 2, and post 3) correspond to T1, the first three days after the inversion. P5, P6, and P7 indicate T2, the fifth, sixth, and seventh days after the inversion. [file JPI-77-e70057-s004.tiff]

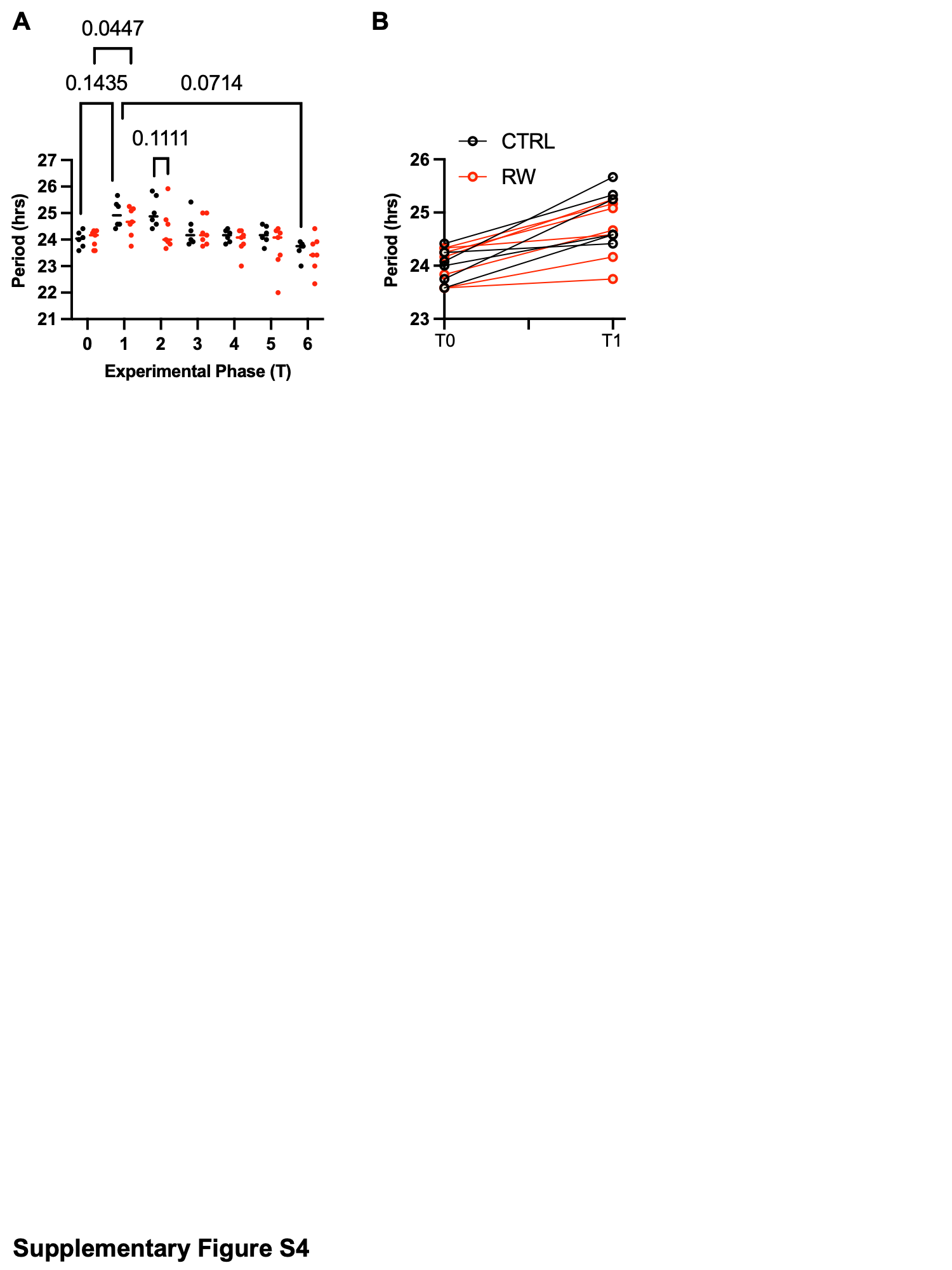

Supplement: Supplementary file 4 — Supporting Figure S4. Related to Table 2. (A,B) Period values determined by autocorrelation for individual mice throughout the experiment (A) or in experimental phases T0 and T1 showing the change for each animal (B). Black symbols and lines depict mice maintained in cages without running wheels throughout the experiment (CTRL); red symbols and lines depict mice that were provided running wheels after the light‐dark inversion (RW). [file JPI-77-e70057-s002.tiff]
